# Flow Cytometric Pattern of TCRVδ Subtype Expression Rapidly Identifies γδT Cell Lymphoma

**DOI:** 10.3389/fonc.2020.00844

**Published:** 2020-06-16

**Authors:** Xiao Chen, Sishu Zhao, Lu Liu, Chun Qiao, Yan Wang, Lei Fan, Huimin Jin, Yujie Wu

**Affiliations:** ^1^Department of Hematology, Jiangsu Province Hospital, The First Affiliated Hospital of Nanjing Medical University, Nanjing, China; ^2^Key Laboratory of Hematology of Nanjing Medical University, Nanjing, China; ^3^Collaborative Innovation Center for Cancer Personalized Medicine, Nanjing, China

**Keywords:** TCR, γδ T cell lymphoma, Vδ1, Vδ2, flow cytometry

## Abstract

**Background:** γδT cell lymphoma (γδ TCL) is a class of hematopoietic malignancy that expresses the γδ T cell receptor (TCR) with a low incidence. Determining the clonal proliferation of γδT cells is important for the diagnosis of such malignancies. Few studies have used flow cytometry to detect VδTCR and its subtypes (Vδ1 and Vδ2) at the protein level, although it is a practical method for determining the neoplastic γδT cells.

**Methods:** A TCRVδ-based 10-color protocol was designed for the detection of malignant proliferation of γδT subtype cells by multiparameter flow cytometry, and the diagnostic results were compared with the gene rearrangement results.

**Results:** All 19 cases of γδ TCL were positive for cluster of differentiation 3 (CD3) and TCR γδ and presented with abnormal distribution patterns of Vδ1 and Vδ2, of which 16 of the 19 cases showed a restricted Vδ1 staining pattern and the remaining three cases lacked the expression of either Vδ1 or Vδ2. Among the 10 normal controls and 11 patients with reactively higher CD4 and CD8 double-negative ratio, the percentage of Vδ2 positive events (range: 16.4–99.0%) was significantly higher than that of Vδ1 (range: 0–50.5%; *p* < 0.0001), and all cases had a normal Vδ distribution pattern. To detect clonality, there was no difference in the detection rate between the TCRVδ analysis and the gene scanning techniques (*p* = 1.000) with a high degree of coincidence (Kappa = 0.850, *p* < 0.001). The heteroduplex analysis was less sensitive than the other methods but was more specific (100%) than the gene scanning techniques, and the TCRVδ subtype analysis had the highest sensitivity, specificity, positive predictive value, and negative predictive value. Compared with molecular methods, immunophenotyping is able to distinguish the T cell lineage.

**Conclusion:** The γδT panel, based on the TCRVδ antibody by flow cytometry, could be advantageous for the rapid identification of suspected γδTCL.

## Introduction

Mature T cell tumors are a group of tumors characterized by the proliferation of T cell malignant clones, accounting for ~12% of lymphoid tumors worldwide (1). There is increasing evidence that immunophenotypic and molecular features are important for the diagnosis and the treatment of lymphoma. The 2016 World Health Organization classification criteria for lymphoma also emphasize the importance of combining morphology, immunophenotyping, and molecular features (2). The application of immunophenotyping and gene expression profiling has greatly enhanced the understanding of the pathogenesis of B-cell lymphoma in recent years. However, due to the complexity of the T cell system, some atypical T cells may have reactive proliferation, so it is often difficult to determine whether T cells are malignant clones (3).

The surface of mature T cells has a molecular structure that specifically recognizes and binds to the antigen, called T cell receptor (TCR). The TCR is a dipeptide chain molecule with two heterodimers: αβTCR (TCR-1) and γδTCR (TCR-2). According to different TCRs, the T cells can be divided into two different subtypes of αβ T cells and γδ T cells (4). Unlike αβ T cells, the γδ T cells account for only 1–5% of peripheral blood lymphocytes and are mainly divided into two subgroups of Vδ1 and Vδ2. Vδ1 accounts for 10–50% of γδT cells and is mainly distributed in epithelial tissues; the Vδ2 cells are mainly present in peripheral blood because the Vδ2 chain of TCR is often paired with Vγ9, also known as Vγ9Vδ2 T cells, which account for more than 90% of peripheral blood γδT cells (5, 6). Studies have reported that Vδ1 cells are significantly increased in the peripheral blood and the tissues of patients with pulmonary sarcoidosis, mycobacterium leprae infection, multiple sclerosis, and hematopoietic stem cell transplantation. The oligoclonal amplification of Vδ1 cells has been found by sequencing the complementarity-determining region 3 structure of the TCR-delta chain (7).

γδT cell lymphoma (γδ TCL) is a class of malignant hematopoietic tumors that express γδ TCR with a low incidence. The tumor cells originate from a small group of T cells with a precursor-like phenotype, but this group of T cells is not well-defined (8). Determining the clonal proliferation of γδT cells is important for the diagnosis of such malignancies, and immunophenotyping and molecular detection techniques are important tools. The γδ TCL expresses cluster of differentiation 3 (CD3) and does not express CD4 and CD8, although a small number of cases express CD8 at low levels (9). The use of flow cytometry to detect γδTCR and its subtypes (Vδ1 and Vδ2) at the protein level is a practical method for determining the clonality of γδT cells. TCR gene rearrangement is a molecular method for detecting the clonal proliferation of T cells, including heteroduplex analysis and gene scanning techniques as well as gradually substituted Southern blot analysis (10, 11). However, the genetic detection techniques which only provide indirect evidence of clonality are complex and time consuming, and their application to routine detection is limited.

In this study, we designed a TCRVδ-based 10-color protocol for the detection of malignant proliferation of γδT subtype cells by multiparameter flow cytometry and compared their diagnostic results with the gene rearrangement results (heteroduplex analysis and gene scanning techniques) in a group of definite diagnostic γδ TCL. Finally, the value of TCRγδ subset panel for the diagnosis of γδ TCL was evaluated. Our study represents the first attempt to assess the clonal proliferation of TCRVδ subsets by flow cytometry that will allow us to diagnose γδTCL.

## Materials and Methods

### Case Identification

**Figure 1:**
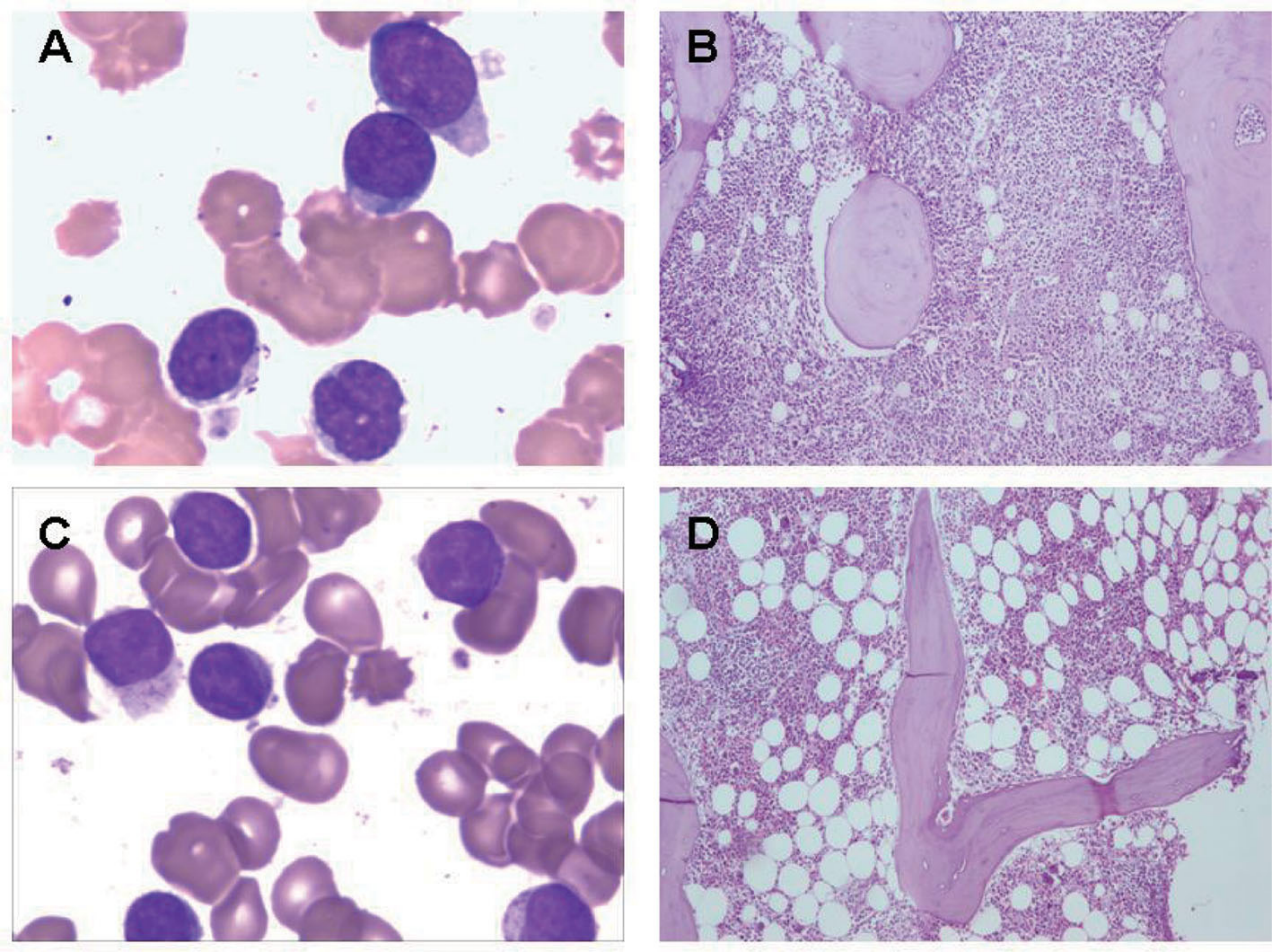
Bone marrow findings in γδ TCL. **(A)** Bone marrow aspirate smear from a patient with γδ TCL. The neoplastic cells showed medium-sized with irregular nuclear contours and hyperchromatic nuclei. **(B)** Bone marrow biopsy from a patient with γδ TCL. The neoplastic lymphocytes showed diffuse hyperplasia. **(C)** Bone marrow aspirate smear from a patient with γδ T-LGL leukemia. The neoplastic cells showed large granular lymphocytes with azurophilic granules. **(D)** Bone marrow biopsy from a patient with γδ T-LGL leukemia. The neoplastic lymphocytes showed small amount of foci.

Patients who were admitted to the Jiangsu Province Hospital (Jiangsu, China) from January 2018 to July 2019 were enrolled in the study. Comprehensive clinical data (e.g., clinical symptoms, pathology reports, laboratory tests, and imaging studies) from all patients were collected and divided into two categories: definitive diagnosis of TCRγδ TCL (19 cases) and non-T cell malignancy tumor (with a reactively higher CD4 and CD8 double-negative ratio; 11 cases). The characteristics of the 19 patients with mature T cell lymphoma are summarized in Table 1. According to the 2016 WHO diagnostic criteria combined with clinical, morphological, immunological, and molecular biology, eight cases were diagnosed with T cell large granular lymphocytic (T-LGL) leukemia, five cases were diagnosed with hepatosplenic T cell lymphoma (HSTCL), and the remaining six cases belonged to other types of TCRγδ+ peripheral T cell lymphoma (PTCL). There were eight males and 11 females with a median age of 44 years (range: 7–85 years). A total of eight patients (42%) had B symptoms, 10 (53%) had hepatomegaly, 12 (63%) had splenomegaly, one had a lymph node disease (5%), and nine (47%) had pure red cell aplasia. The median absolute lymphocyte count was 1.87 × 10^9^/L (range: 0.64–9.16 × 10^9^/L), and lymphocytosis (>4 × 10^9^/L) was identified in five (26%) patients. The median neutrophil count was 1.28 × 10^9^/L (range: 0.48–4.39 × 10^9^/L); nine (47%) patients had neutropenia (<1.5 × 10^9^/L). The median platelet count was 184 × 10^9^/L (range: 52–313 × 10^9^/L); four (21%) patients had thrombocytopenia (<100 × 10^9^/L). The median hemoglobin was 74 g/L (range: 31–150 g/L), and anemia (<100 g/L) was identified in 15 (79%) patients. The β2-MG was >3.0 mg/L in six cases (32%) and was <3.0 mg/L in 13 cases (68%). The neoplastic lymphocytes in the bone marrow and the peripheral blood were all determined by smears or biopsy (Figure 1). The extent of bone marrow or peripheral blood involvement was >20% in 10 cases (53%) and <20% in nine cases (47%). The neoplastic cells showed large granular lymphocytes in γδ T-LGL leukemia and medium size and variable morphology in other types of PTCL, including HSTCL. Increased LGLs were observed in two of seven cases with γδ T-LGL leukemia. A total of 11 patients with a reactively higher CD4 and CD8 double-negative ratio were also included in the study, including three cases of autoimmune diseases, one case of B-cell lymphoma/leukemia, one case of aplastic anemia, one case of Epstein–Barr virus infection, one case of unexplained lymphadenopathy, and four cases of undefined non-T cell tumor. The median age was 31 years (range: 20–77 years), including six males and five females.

**Table 1:** Clinicopathologic Features of 19 patients with γδ TCL.

**Clinical characteristics**	**n**	**%**
Sex		
Male	8	42
Female	11	58
**Age (years)**		
Median	44	
Range	7–85	
**Type**		
γδ + T-LGL leukemia	8	42
γδ + HSTCL	5	26
another γδ?TCL	6	32
**B-symptoms**		
Present	8	42
Absent	11	58
**Splenomegaly**		
Present	12	63
Absent	7	37
**Hepatomegaly**		
Present	10	53
Absent	9	47
**Lymphadenopathy**		
Present	1	5
Absent	18	95
**BM or PB involvement (%)**		
>20	10	53
≤20	9	47
**β2-MG**		
>3.0 mg/L	6	32
≤3.0 mg/L	13	68
**Absolute lymphocyte count (× 10^9^/L) 9/L)**		
Median	1.87	
Range	0.64–9.16	
Above 4 × 10^9^/L	5	26
**Platelet (×10^9^/L)**		
Median	184	
Range	52–313	
Below 100 × 10^9^/L	4	21
**Absolute neutrophils count (× 10^9^/L)**		
Median	1.28	
Range	0.48–4.39	
Below 1.5 × 10^9^/L	9	47
**Hemoglobin (g/L)**		
Median	74	
Range	31–150	
Below 100 g/L	15	79
**PRCA**		
Present	9	47
Absent	10	53

A total of 19 patients with γδT cell tumors and 11 patients with a reactively higher CD4 and CD8 double-negative ratio were collected for lymphoid proliferative disease panel screening for fresh bone marrow, peripheral blood, or tissue samples. All 30 cases and 10 normal controls were screened by TCRγδ panel staining, followed by routine flow analysis, and 28 cases (17 cases with γδTCL and 11 cases with a reactively higher CD4 and CD8 double-negative ratio) were subjected to TCR gene rearrangement detection. The specimen types include 23 bone marrow specimens, 16 peripheral blood specimens, and one spleen specimen. All the patients provided written informed consent to have their samples included in the study, and approval was also obtained by the hospital ethics committee.

### Histologic Assessment and Flow Cytometry Analysis of the TCR γδ Panel

The bone marrow and peripheral blood specimens were evaluated at initial diagnosis. Aspirate smears and biopsy specimens were stained with Wright–Giemsa and hematoxylin and eosin, respectively. A volume of 2 ml of bone marrow or peripheral blood from the patient was collected, and heparin was anticoagulated and detected within 24 h. The fresh spleen specimen was ground into mononuclear cells. The TCRγδ panel combination was CD3 (UCHT-1)/CD4 (13B8.2)/CD8 (B9.11)/HLA-DR (Immu-357)/CD45 (J.33)/TCRαβ (IP26A)/TCRγδ (IMMU510)/TCRVδ1 (R9.12)/TCRVδ2 (IMMU389), and the corresponding labeled fluorescein was APC-A750, APC, Alexa Fluor700, ECD, KRO, PE, FITC, PC7, and PB. A variable panel of antibodies was also used at di?erent institutions; most panels included Kappa, Lambda, CD45, CD19, CD16, CD56, CD2, CD7, CD5, CD57, CD1a, and CD10. All the antibodies were obtained from Beckman Coulter, Inc. (Brea, CA, USA). All specimens, including normal controls, were incubated with the labeled antibody for 15 min at room temperature in the dark. After incubation, a lysing solution (Beckman Coulter) was used to lyse the red blood cells, and the cells were washed with phosphate-buffered saline (PBS), centrifuged, and then re-suspended in 500 μl PBS, after which 50,000 cells were collected on the Navios flow cytometer (Beckman Coulter). Antigen expression in the CD3+ cell population was analyzed by Kaluza version 2.1 (Beckman Coulter), and the distribution of γδT cell subsets was determined by logicle scale histograms. When the target cell-expressed TCRγδ+ and Vδ1 had restricted expression or Vδ1/Vδ2 was negatively expressed, it was considered that the γδT cells had clonal proliferation in the specimen.

### TCR Gene Rearrangement Analysis

Peripheral blood or bone marrow mononuclear cells were isolated using Ficoll-Hypaque. DNA extraction and purification were performed according to the DNA extraction instructions provided by the QIAamp Blood Kit (Qiagen, Hilden, Germany). The PCR amplification of housekeeping genes was performed by agarose gel electrophoresis to quantify and determine the DNA template integrity. A total of six groups were used to amplify the TCR gene corresponding fragments, TCRβ was divided into three groups (TCR tube A–C), TCR γ was divided into two groups (TCR γ tube A–B), and TCRδ was divided into one group. Amplification was performed with an Applied Biosystems Veriti Thermal Cycler (Framingham, MA, USA). The reaction conditions were 95°C for 7 min initially, followed by 95°C for 30 s, 60°C for 45 s, and extension at 72°C for 90 s. A total of 35 cycles were conducted, final extension was extended to 10 min, and the products were stored at 15°C. The samples with known monoclonal TCR rearrangements were used as positive controls, and normal human mononuclear cells were used as negative controls. Amplification was conducted under the above-mentioned conditions. The PCR products of each tube were subjected to heteroduplex analysis and gene scanning analysis, respectively. The samples were detected by 8% polyacrylamide gel (Anamed Gross-bieberau, Germany) with electrophoresis at 180 V for ~30 min. The gels were stained with ethidium bromide for 3–6 min and analyzed on a BIO-RAD gel imager (BIO-RAD, USA). The gene scanning analysis was performed using a 3,500 Dx gene analyzer (Applied Biosystems, USA) after the PCR products were mixed with formamide and Genescan-500LIZ (Applied Biosystems, USA). The reaction conditions were 95°C for 2 min and 4°C for 5 min.

### Statistical Analysis

Data analysis was performed using SPSS statistical analysis software (version 23.0). The mean percentages of TCRVδ-positive events between the two groups were compared using Student's *t*-test. Comparisons of the count data between groups were performed by McNemar, Kappa tests, χ^2^ test, or Fisher's exact test. *p* < 0.05 was considered as statistically significant.

## Results

### TCRVδ Subtype Analysis by Flow Cytometry

**Figure 2:**
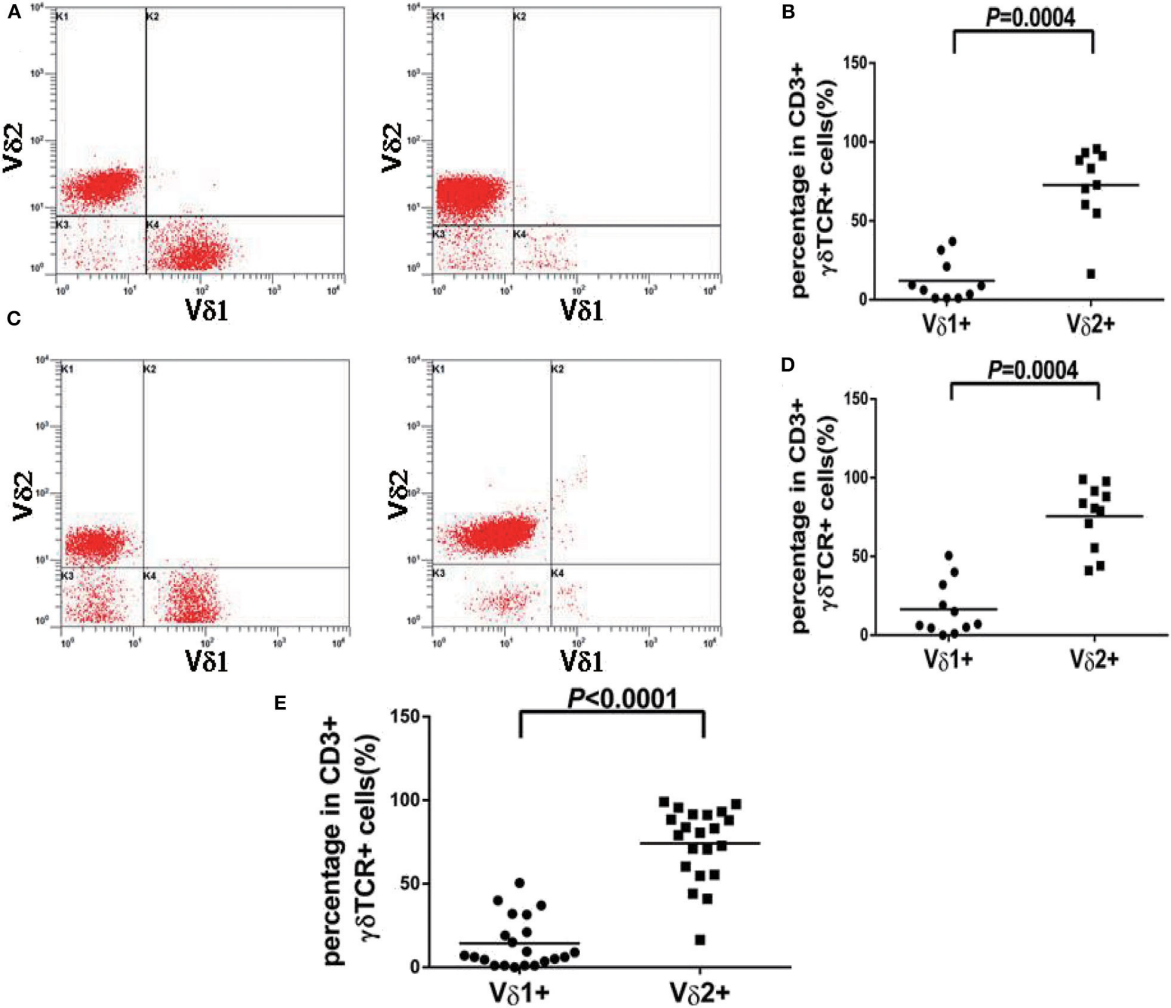
Vδ1 and Vδ2 expression pattern of γδT cell from normal controls and patients with reactively higher CD4 and CD8 double-negative ratio. **(A)** Representative peripheral blood samples from normal controls showed the normal distribution pattern of Vδ1 and Vδ2 in healthy individuals. **(B)** The percentages of Vδ2 positive events were significantly higher than Vδ1 in normal controls (*p* = 0.0004). **(C)** Representative peripheral blood samples or bone marrow samples from patients with reactively higher CD4 and CD8 double-negative ratio showed the normal distribution pattern of Vδ1 and Vδ2. **(D)** The percentages of Vδ2 positive events were significantly higher than Vδ1 in patients with reactively higher CD4 and CD8 double-negative ratio (*p* = 0.0004). **(E)** The percentages of Vδ2 positive events were significantly higher than Vδ1 in all 21 cases without TCL (*p* < 0.0001).

**Figure 3:**
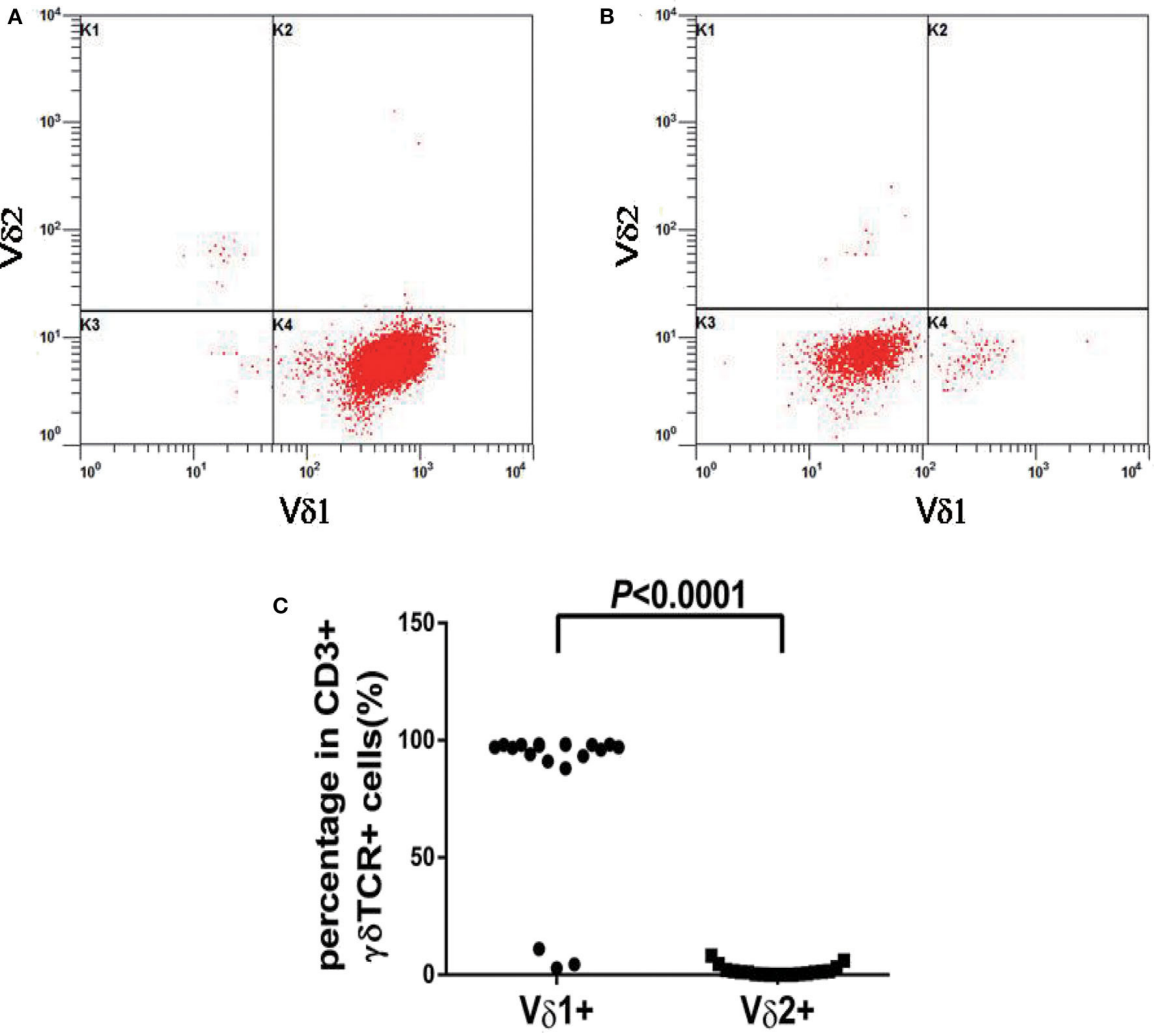
Vδ1 and Vδ2 expression patterns of γδT cells from patients with γδ TCL. **(A)** A restricted Vδ1 staining pattern were detected in 16 of 19 cases. **(B)** The neoplastic cells in three cases showed absent expression in both Vδ1 and Vδ2. **(C)** The percentages of Vδ1 positive events were significantly higher than Vδ2 in γδ TCL (*p* < 0.0001).

**Figure 4:**
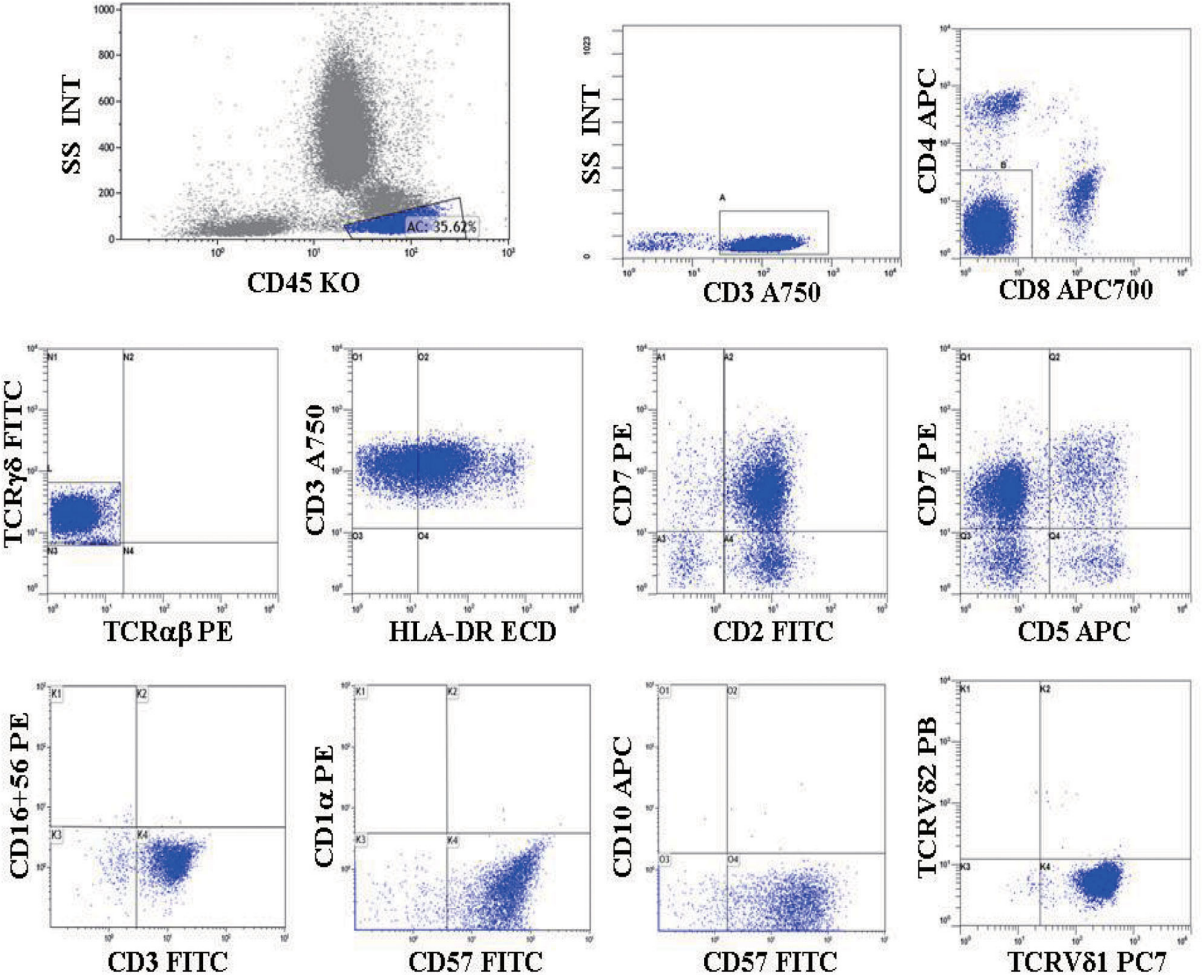
(Case 15) Flow cytometric immunophenotyping of γδ TCL. A distinct CD4–/CD8– T cell population was detected and was positive for CD3, TCRγδ, CD2, CD7, CD57, HLA-DR, and TCR Vδ1, but negative for TCRαβ, CD5, CD16, CD56, CD1a, CD10, and TCR Vδ2.

In our study, 19 cases of γδ TCL were diagnosed based on clinical symptoms, pathological reports, laboratory tests, and imaging examinations, and 11 cases with reactively higher CD4 and CD8 double-negative cells eventually showed no evidence of T cell malignancy. The immunophenotypes of these 19 cases are shown in Table 2. All were positive for CD3 and TCR γδ but negative for CD4 and CD8 or only partially positive for CD8 (case 4). CD2, CD7, and CD5 were analyzed by flow cytometry in 18 cases. CD2 was positive in 17 of 18 cases (94.4%), CD7 was positive in 18 of 18 cases (100%), and CD5 was positive in five of 18 cases (27.8%), of which case 6 showed a weak expression of CD2, CD7, and CD5 and case 17 showed a weak expression of CD5. The expression of Vδ1 and Vδ2, members of the γδ TCR family, was also analyzed by flow cytometry. To evaluate the distribution pattern of Vδ1 and Vδ2 in normal controls, we collected peripheral blood from 10 healthy individuals. As shown in Figures 2A,B, all detectable CD3+γδ T cell subsets had a Vδ expression pattern with the extent of Vδ1 ranging from 1.0 to 37.1% and that of Vδ2 ranging from 16.4 to 95.6%, indicating a superior expression of Vδ2 in the normal controls (*p* = 0.0004). Among the 11 patients with reactively higher CD4 and CD8 double-negative cells, the expression of subtypes of γδ TCR (Vδ1 and Vδ2) was also detected by flow cytometry. The detectable γδ T cell subsets in all 11 cases had the same Vδ expression pattern as that of normal controls. The percentage of Vδ2 positive events (range: 41.0–99.0%) was significantly higher than that of Vδ1 (range: 0–50.5%; *p* = 0.0004). The immunophenotypic characteristics of the CD3+γδ T cell subsets in specimens with a reactively higher CD4 and CD8 double-negative ratio are shown in Figures 2C,D. Overall, all γδ T cell subsets in the 21 cases without TCL had a higher percentage of Vδ2, ranging from 16.4 to 99.0%, than that of Vδ2, ranging from 0 to 55.5% (*p* < 0.0001) (Figure 2E). Among the 19 patients with γδ TCL, the percentages of Vδ1-positive events were significantly higher than those of the Vδ2-positive events (*p* < 0.0001). In 16 of 19 cases (84.2%), the γδ T neoplastic cells had a high percentage of Vδ1 expression (range: 88.0–98.4%), which indicated a restricted Vδ1 expression pattern. The neoplastic cells in the remaining three cases showed absent expression in both Vδ1 and Vδ2 (Figure 3). In addition, both of the expression patterns of the subsets of γδ T cells demonstrated a monoclonal proliferation of γδ T cells. Notably, the γδT cells of case 2 were detected again after treatment, and Vδ1 and Vδ2 were found to have a normal biphasic distribution pattern. Natural killer (NK) cell-associated markers, CD56, CD16, and CD57, were also determined in seven cases. CD56 was positive in three of seven cases (42.9%), CD16 in two of seven cases (28.6%), and CD57 in three of seven cases (42.9%). In addition, CD1a and CD10 were detected in two patients (cases 7 and 15), both of whom showed negative expression. The results of immunophenotyping by flow cytometry of a representative case (case 15) are shown in Figure 4.

**Table 2:** Immunophenotype in 19 Cases of γδ TCL.

**No. of Cases**	**Flow cytometry phenotype**											
	**CD3 CD4**	**CD8**	**CD2**	**CD7**	**CD5**	**TCRαβ**	**TCRγδ**	**Vδ1**	**Vδ2**	**CD56**	**CD57**	**CD16**
1	+ –	–	+	+	–	–	+	–	–	ND	ND	ND
2	+ –	–	+	+	–	–	+	+	–	+	–	+
3	+ –	–	+	+	–	–	+	+	–	ND	ND	ND
4	+ –	dim+	+	+	–	–	+	+	–	+	+	–
5	+ –	–	+	+	+	–	+	+	–	+	–	+
6	+ –	–	dim+	dim+	dim+	–	+	–	–	ND	ND	ND
7	+ –	–	–	+	+	–	+	+	–	–	–	–
8	+ –	–	+	+	-	–	+	-	–	ND	ND	ND
9	+ –	–	ND	ND	ND	–	+	+	–	ND	ND	ND
10	+ –	–	+	+	–	–	+	+	–	ND	ND	ND
11	+ –	–	+	+	–	–	+	+	–	ND	ND	ND
12	+ –	–	+	+	+	–	+	+	–	ND	ND	ND
13	+ –	–	+	+	–	–	+	+	–	ND	ND	ND
14	+ –	–	+	+	–	–	+	+	–	ND	ND	ND
15	+ –	–	+	+	–	–	+	+	–	–	+	–
16	+ –	–	+	+	–	–	+	+	–	ND	ND	ND
17	+ –	–	+	+	dim+	–	+	+	–	–	+	–
18	+ –	–	+	+	–	–	+	+	–	–	–	–
19	+ –	–	+	+	–	–	+	+	–	ND	ND	ND

*TCR, T-cell receptor; +, positive; –, negative; ND, not done*.

### Molecular Analysis

**Figure 5:**
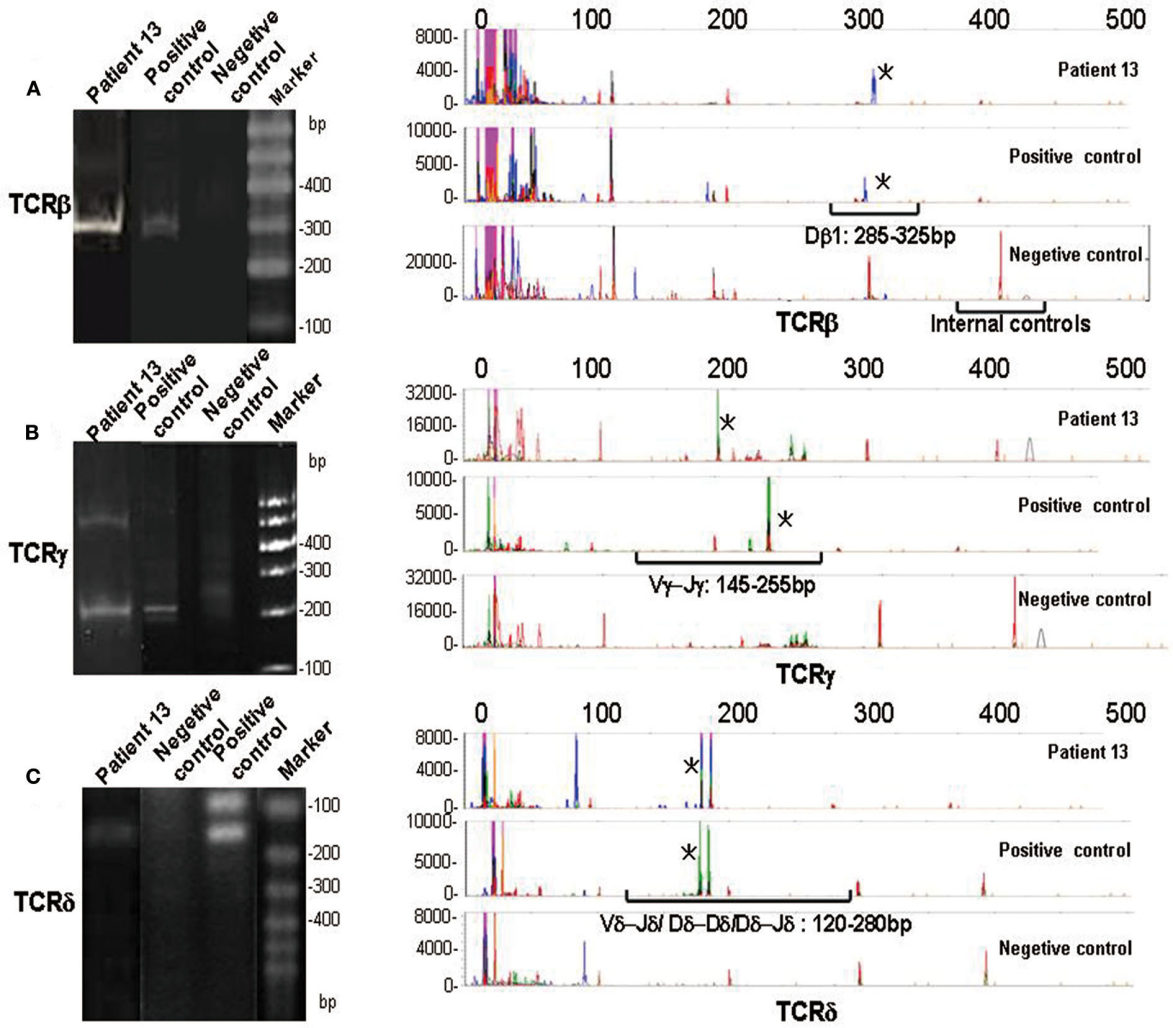
PCR analysis of TCR gene rearrangements. Heteroduplex analysis (left panel) and GeneScanning (right panel) of patient 13, negetive controls, and positive controls. The negetive controls show a vague smear in heteroduplex analysis and a complex peak pattern in GeneScanning. The red peaks indicate housekeeping genes (internal controls). The positive controls and patient 13 show visible bands in heteroduplex analysis and monoclonal peaks (marked with asterisk) in GeneScanning in approximate position of molecular weight. **(A)** Analysis of TCRβ gene rearrangements (monoclonal peaks are located in Dβ1 gene segments: 285–325 bp). **(B)** Analysis of TCRγ gene rearrangements (monoclonal peaks are located in Vγ- Jγ gene segments: 145–255 bp). **(C)** Analysis of TCRδ gene rearrangements (monoclonal peaks are located in Vδ-Jδ/Dδ- Dδ/Dδ- Jδ gene segments: 120–280 bp).

Gene scanning was used to analyze the TCR gene rearrangement in 17 patients with γδ TCL and 11 cases with a reactively higher CD4 and CD8 double-negative ratio. It was found that 16 of 17 (94.1%) cases diagnosed with TCL had at least one TCR gene rearrangement. Among the 11 patients with reactively higher CD4 and CD8 double-negative cells, 10 cases were negative for rearrangement. According to the primer design principle of the BIOMED-2 guidelines (11), there are 59 primers for TCR gene cloning analysis. TCRβ, TCRγ, and TCRδ were detected by a primer combination. TCRβ is divided into three groups: groups A–C. The primers included 23 Vβ, 2 Dβ, and 13 Jβ, and the detection of Vβ-Jβ and Dβ-Jβ was completed. The TCRγ assay was divided into groups A and B, including three Vγ and two Jγ primers. The TCRδ detection primers included six Vδ, four Jδ, and six Dδ. In the process of T cell differentiation, δ gene rearrangement occurs first, followed by TCRγ, TCRβ, and TCRα gene rearrangement. The different gene rearrangement patterns are of great value for disease diagnosis. The combination of TCRβ and TCRγ tubes can detect all clonal T cells, and the TCRδ tube has an important diagnostic value for TCRγδ T cell clonal proliferation. The results of the TCR gene rearrangement in 17 patients with T cell tumors are shown in Table 3. The detection rates of TCRβ, TCRγ, and TCRδ tubes were 64.7, 70.6, and 83.3%, respectively. Among the three groups of TCRβ, the positive rates of TCRβ-A, TCRβ-B, and TCRβ-C were 0, 5.8, and 58.8%, respectively. The TCRγ-A tube had the most sensitive reaction rate at 70.6%, and TCRγ-B had a detection rate of 41.2%. In TCR gene rearrangement with gene scanning technique, the TCRβ, TCRγ, and TCRδ rearrangements were all positive in five of the 17 cases (29.4%). The results of the TCR gene rearrangement of a representative case (case 13) are shown in Figure 5.

**Table 3:** TCR gene rearrangement by gene scanning technique in patients with γδ TCL.

**No. of cases**	**TCRβ**			**TCRγ**		
	**TCRβ-A**	**TCRβ-B**	**TCRβ-C**	**TCRγ-A**	**TCRγ-B**	**TCRδ**
1	–	–	+	–	–	ND
2	–	–	+	–	–	ND
3	–	–	–	+	+	+
4	–	+	–	+	–	–
5	–	–	–	+	+	+
6	–	–	–	–	–	–
7	–	–	+	–	–	ND
8	–	–	+	+	+	ND
9	–	–	–	+	+	+
10	–	–	+	+	–	+
11	–	–	–	–	–	+
12	–	–	+	+	–	+
13	–	–	+	+	+	+
14	–	–	+	+	–	ND
15	–	–	+	+	+	+
16	–	–	+	+	–	+
17	–	–	–	+	+	+
18	ND	ND	ND	ND	ND	ND
19	ND	ND	ND	ND	ND	ND

*“+”, a dominant T cell clone; ND, not done*.

### Sensitivity and Specificity of TCRVδ Subtype Analysis and TCR Gene Rearrangement Analysis

First, Kappa test and McNemar test were used to evaluate the degree of coincidence between TCRγδ analysis and TCR gene rearrangement (gene scanning analysis). The results showed that there was no difference in the detection rate between the two methods (*p* = 1.000), and the degree of coincidence was high (Kappa = 0.850, *p* < 0.001) (Table 4). Next, we evaluated the sensitivity, specificity, and positive predictive value (PPV) and negative predictive value (NPV) of TCRVδ subtype analysis, heteroduplex analysis, and gene scanning analysis, and the results are summarized in Table 5. A total of 28 specimens included 17 T cell tumors and 11 patients with reactively higher CD4 and CD8 double-negative cells. The sensitivity of heteroduplex analysis and gene scanning analysis in detecting clonality was 53 and 94%, respectively. Heteroduplex analysis was less sensitive than the other methods but was more specific than gene scanning analysis (100%). For the diagnosis of TCL, we found that TCRVδ1 and TCRVδ2 analysis by flow cytometry for detecting clonality had the highest sensitivity, specificity, PPV, and NPV.

**Table 4:** The degree of coincidence between TCRγδ analysis and TCR gene rearrangement.

**Parameter**	**Number of patients**		
	**TCR gene rearrangement (gene scanning analysis)**		
Clonal TCR Vδ (*n* = 28)	Positive	Negative	McNemar test, *p* =1.000;
Positive	16	1	Kappa = 0.850, *p* < 0.001
Negative	1	10	

**Table 5:** Sensitivity, specificity, PPV, and NPV of TCRγδ analysis, heteroduplex analysis, and gene scanning analysis in 17 patients with γδ TCL and 11 patients with reactively higher CD4 and CD8 double-negative ratio.

	**Sensitivity**	**Specificity**	**PPV**	**NPV**
TCR Vδ panel	100% (17/17)	100% (11/11)	100% (17/17)	100% (11/11)
Gene scanning analysis	94% (16/17)	91% (10/11)	94% (16/17)	91% (10/11)
Heteroduplex analysis	53% (8/17)	100% (11/11)	100% (8/8)	55% (11/20)

## Discussion

γδ TCL is a type of peripheral TCL that is rare in clinical practice and has a low incidence. Since Farcet et al. reported the pathological types of hepatosplenic γδTCL in 1990, there have been related reports mainly including hepatosplenic γδ TCL and primary cutaneous γδ TCL (12). Recently, there have been reports on γδ T cell large granular lymphocytic (T-LGL) leukemia, although most T-LGL leukemia cases belong to the αβTCR type (13–15). Compared with αβ TCR-type T-LGL leukemia, γδ-type T-LGL leukemia patients have more obvious thrombocytopenia, lower absolute neutrophil count, and higher CD4 and CD8 double-negative ratios (16). From the immunophenotype, γδT cell tumors often express CD2, CD3, CD7, CD56, and TCRγδ and do not express CD4, CD8, CD5, and TCRαβ (17, 18). Reports on γδ type lymphoma are still rare and have not been thoroughly studied. Our present study, which describes 19 cases of γδ TCL, is the first study to diagnose γδTCL by multiparameter flow cytometry.

The present study analyzed 19 cases of γδ TCL identified in our institution between 2018 and 2019. Similar to the immunophenotype γδ TCL reported in the literature, our data showed that the 19 cases were positive for CD3 and TCR γδ but were negative for CD4 and CD8 or only partially weakly positive for CD8. The conventional antigen expression patterns of γδ TCL are CD3+, CD2+, CD7+/-, and CD5-, and most cases express NK cell antigen CD56, which may express CD16 but not CD57 (17). In our study, CD2 and CD7 were expressed nearly in all cases (17/18 and 18/18, respectively), and CD5 was negative in the vast majority of cases (13/18). CD56 was positive in three of seven (42.9%), CD16 in two of seven (28.6%), and CD57 in three of seven (42.9%) cases. Our findings further confirm that γδTCL showed a similar expression pattern of T cell-associated antigens and a uniform phenotype of NK cell-associated antigens. However, the cases described in our study are too small to summarize definitive conclusions on this issue. The development of various antibodies for different TCR chain domains has made it possible to detect malignant clones of Vβ, Vγ, and Vδ (10, 19). Of importance is that the T-antigen panel of the present study was used to further analyze the distribution characteristics of γδ TCR subpopulations, and the detection of abnormal distribution was helpful to determine the clonal proliferation of γδ T cells, which was beneficial to the diagnosis of CD3+γδ TCL. Previous studies have reported that Vδ1+ cells represented a minority of the TCRγδ+ population (0–30%), while Vδ2 represented the majority (>80%) in normal peripheral blood (20, 21). In agreement with previous documents, the γδ T cell subsets demonstrated a predominance of Vδ2-positive events in either the normal control group or the patients with a reactively higher CD4 and CD8 double-negative ratio, indicating a normal Vδ expression pattern in all cases (5, 20, 21). Thus, the specificity of detecting suspected CD3+γδ TCL by TCR Vδ labeling is 100%. These data support the conclusion that benign γδ T cells express the Vδ2 TCR predominantly. According to the structure of gene V, normal γδT cells are mainly composed of the two subgroups Vδ1 and Vδ2. Vδ2 is expressed in almost all human tissues, which far exceeds Vδ1, which is mainly expressed in the lymph nodes, skin, and tonsils. γδT cells in healthy peripheral blood mainly express Vδ2 TCR, which plays a role in recognizing small-molecule phosphorylated antigens, whereas Vδ1 is mainly expressed in the gastrointestinal tract and is able to recognize stress-induced MHC antigens MIC-A and MIC-B (3, 22). It has been reported that Vδ1 cells clonally proliferate in tumor-infiltrating lymphocytes, which may have an immunosuppressive function (23). Through staining of Vδ1 and Vδ2 antibodies in γδ TCR panel, the γδTCR subpopulations in all 19 cases of CD3+γδ T cell tumors were abnormally distributed. Among them, 16 cases had a restricted Vδ1 staining pattern, and the other three cases were negative for Vδ1 and Vδ2, indicating the expression of an unidentified V element. Both distribution patterns were considered to have T cell malignant proliferation. This is consistent with a report that most γδ T cell malignancies express Vδ1 or other non-Vδ2γδ TCR subpopulations (10). Overall, the sensitivity of determining clonality by detecting the γδ TCR subpopulation was 100%. This implies that immunophenotyping by flow cytometry in bone marrow or peripheral blood specimens is a satisfactory method to identify the neoplastic γδT cells, by detecting the abnormal distribution of the γδ TCR subpopulation. Notably, a patient with γδTCL (case 2) showed a normal biphasic distribution pattern of Vδ1 and Vδ2 after effective treatment, suggesting that the panel of TCR Vδ subtypes may be used for the therapeutic monitoring of γδTCL. However, to date, few studies have evaluated Vδ1TCR and Vδ2TCR in hematological tumors, which need further investigation.

The detection of TCR gene rearrangement by molecular biology methods plays an important role in the diagnosis of γδTCL. This study showed that 16 of 17 cases diagnosed with TCL detected at least one TCR gene rearrangement with a sensitivity of 94%. In addition, the detection rates of TCRβ, TCRγ, or TCRδ tubes were 64.7, 70.6, and 83.3%, respectively. The TCRδ chain biallelic rearrangement of γδ T cells as detected with PCR or Southern blot analysis has an important diagnostic value for γδTCL. So, it is often difficult to diagnose γδ-type TCL on molecular methods alone due to the relatively low positive rate of the TCRδ gene. However, many γδ-type TCLs present with TCRβ chain gene rearrangements, and similarly, most αβ-type TCLs also present with clonal rearrangements of the TCRγ gene. In fact, determining TCRβ or TCRγ rearrangement to distinguish whether the tumor cells are of the αβ type or the γδ type is not possible (10, 11, 24).

The detection of Vδ subtype abnormal expression by flow cytometry is a simple and rapid method for peripheral blood, bone marrow, and tissue samples from patients with γδ TCL. With high sensitivity and specificity, the TCRδ antibody could be adequate for meaningfully identifying γδT cell malignancies at the protein level. Of note is that the detection of TCRδ could be yet more specific than TCRγ antibody in the diagnosis of γδ TCL (6). Compared with antibody-based flow cytometry, the molecular methods for detecting TCR gene rearrangements are more widely used and provide a wealth of information. To detect monoclonal TCR gene rearrangement, the sensitivity and specificity of the Heteroduplex assay in our study were 55 and 100%, respectively. Heteroduplex analysis is a simple, fast, and inexpensive tool for detection of TCR gene rearrangement, but with lower sensitivity and higher false-negative rates. The formation of a heteroduplex may consume a portion of the monoclonal PCR product, so the results are susceptible to the number of polyclonal lymphocytes (25). From the aspect of specificity, heteroduplex analysis is higher, and the false-positive rate is lower. Gene scanning technology, another molecular method for TCR gene rearrangement, is relatively simple, rapid, and sensitive and can detect 0.5–1% of monoclonal T cells, but this method is expensive (11). Recently, advances in next-generation sequencing (NGS) based on T cell repertoire metrics analysis have tremendously increased our knowledge of clonality and diversity in tumors in depth and at high throughput. In addition, NGS is more sensitive and precise for the identification of clonality than the techniques used in the present study (26–28). However, this technique has not been widely accepted for the detection of TCR gene rearrangement clinically due to its high cost. In fact, our data showed that there was no difference in detection rate between the gene scanning technology and the immunophenotyping method (*p* = 1.000), and the degree of coincidence was high (Kappa = 0.850, *p* < 0.001). Despite similar sensitivity and specificity between the two methods, TCR gene rearrangement is limited to finding evidence of clonality of T cells since it is not a marker for identifying the origin of T cell clones. Several studies have reported that several T lymphoid malignancies have different conventional TCR gene rearrangement patterns, called cross-line gene rearrangements (11, 29). The clonality of mature lymphoid malignancies might be detectable at the protein level due to the fact that functionally rearranged TCR genes are expressed on the surface membrane of TCR molecules. Therefore, molecular detection of TCR gene rearrangement can only serve as a reference for the clonality of γδ TCL, and its specific diagnosis should be combined with clinical, morphological, and immunological typing.

In conclusion, we first measured the γδT cell subtype by flow cytometry based on the TCRVδ1 and TCRVδ2 antibody combination for the diagnosis of suspected CD3+ γδT cell malignancies, which is simple, rapid, and highly accurate. Interestingly, flow cytometric analysis on fresh cell suspensions might be an alternative for distinguishing γδ TCL when tissue samples are unavailable, although pathology biopsy is the gold standard. Our search for evidence of T cell clonality showed that it has high sensitivity and specificity when heteroduplex analysis is combined with gene scanning technology. However, compared with immunophenotyping, molecular methods are not able to distinguish the T cell lineage. Remarkably, due to clonal origin occurring in a small number of benign proliferating lymphocytes, some scholars have suggested that molecular clonal evidence is not always equivalent to malignancy. Therefore, the correct diagnosis of γδ TCL should integrate clinical data, morphology, and immunophenotyping with molecular biology.

## Data Availability Statement

The datasets generated for this study are available on request to the corresponding author.

## Ethics Statement

The studies involving human participants were reviewed and approved by Jiangsu Province Hospital. The patients/participants provided their written informed consent to participate in this study.

## Author Contributions

XC and YWu designed the project and wrote the manuscript. XC, YWu, SZ, and LL analyzed the data. CQ and YWa performed the experiments. LF and HJ provided critical comments and suggestions and made the figures. All the authors reviewed the manuscript and approved the submitted version of the manuscript.

## Conflict of Interest

The authors declare that the research was conducted in the absence of any commercial or financial relationships that could be construed as a potential conflict of interest.
